# Differential effects of Down's syndrome and Alzheimer's neuropathology on default mode connectivity

**DOI:** 10.1002/hbm.24720

**Published:** 2019-07-26

**Authors:** Liam R. Wilson, Deniz Vatansever, Tiina Annus, Guy B. Williams, Young T. Hong, Tim D. Fryer, Peter J. Nestor, Anthony J. Holland, Shahid H. Zaman

**Affiliations:** ^1^ Cambridge Intellectual and Developmental Disabilities Research Group, Department of Psychiatry University of Cambridge Cambridge UK; ^2^ Institute of Science and Technology for Brain‐inspired Intelligence, Fudan University Shanghai China; ^3^ The Wolfson Brain Imaging Centre, Department of Clinical Neurosciences University of Cambridge Cambridge UK; ^4^ Queensland Brain Institute, University of Queensland Brisbane Australia

**Keywords:** Alzheimer's disease, anti‐correlation, default mode network, Down's syndrome, functional connectivity, memory

## Abstract

Down's syndrome is a chromosomal disorder that invariably results in both intellectual disability and Alzheimer's disease neuropathology. However, only a limited number of studies to date have investigated intrinsic brain network organisation in people with Down's syndrome, none of which addressed the links between functional connectivity and Alzheimer's disease. In this cross‐sectional study, we employed ^11^C‐Pittsburgh Compound‐B (PiB) positron emission tomography in order to group participants with Down's syndrome based on the presence of fibrillar beta‐amyloid neuropathology. We also acquired resting state functional magnetic resonance imaging data to interrogate the connectivity of the default mode network; a large‐scale system with demonstrated links to Alzheimer's disease. The results revealed widespread positive connectivity of the default mode network in people with Down's syndrome (*n* = 34, ages 30–55, median age = 43.5) and a stark lack of anti‐correlation. However, in contrast to typically developing controls (*n* = 20, ages 30–55, median age = 43.5), the Down's syndrome group also showed significantly weaker connections in localised frontal and posterior brain regions. Notably, while a comparison of the PiB‐negative Down's syndrome group (*n* = 19, ages 30–48, median age = 41.0) to controls suggested that alterations in default mode connectivity to frontal brain regions are related to atypical development, a comparison of the PiB‐positive (*n* = 15, ages 39–55, median age = 48.0) and PiB‐negative Down's syndrome groups indicated that aberrant connectivity in posterior cortices is associated with the presence of Alzheimer's disease neuropathology. Such distinct profiles of altered connectivity not only further our understanding of the brain physiology that underlies these two inherently linked conditions but may also potentially provide a biomarker for future studies of neurodegeneration in people with Down's syndrome.

## 1 INTRODUCTION

Down's syndrome, or trisomy 21, is the most common identified cause of intellectual disability worldwide (Bittles, Bower, Hussain, & Glasson, 2007; Contestabile, Benfenati, & Gasparini, 2010; Dierssen, 2012) and is also strongly associated with the development of amyloidosis—a hallmark of Alzheimer's disease. The amyloid cascade hypothesis (Hardy & Higgins, 1992; Selkoe, 1991) suggests that this increased risk is due to the supernumerary copy of the amyloid precursor protein (APP) gene that is present in Down's syndrome, which in turn is posited to increase the levels of beta‐amyloid (Aβ) present in the brain. Indeed, histological studies have found Aβ plaques in the brains of children and teenagers with Down's syndrome (Lemere et al., 1996; Leverenz & Raskind, 1998), while this neuropathology appears in nearly 100% of people with Down's syndrome over 40 years of age (Mann, 1988). Moreover, studies of people with Down's syndrome using positron emission tomography (PET) and radioligands such as ^11^C Pittsburgh Compound‐B (^11^C‐PiB) and ^18^F Florbetaben have demonstrated that the presence of fibrillar Aβ is detectable in a very high proportion of people by age 50 (Annus et al., 2015; Hartley et al., 2014; Hartley et al., 2017; Jennings et al., 2015; Lao et al., 2016; Lao et al., 2017).

In the typically developing population with Alzheimer's disease, the topography of amyloid deposition is known to overlap substantially with regions that form a large‐scale functional brain network known as the default mode network (DMN; Buckner et al., 2005; Buckner et al., 2009). Encompassing the medial prefrontal cortex, medial temporal lobe structures, posterior cingulate cortex, precuneus and the angular gyri bilaterally (Andrews‐Hanna, Smallwood, & Spreng, 2014), the regions that form this network have been reported to show a high degree of both functional and structural connectivity (Horn, Ostwald, Reisert, & Blankenburg, 2014) with notably high levels of metabolic activity during resting state conditions in healthy participants (Gusnard, Raichle, & Raichle, 2001). In patients with amnesic symptoms, however, PET imaging studies using ^18^fluorodeoxyglucose (^18^FDG) have shown that specific regions within this network, including the posterior cingulate and medial temporal lobe structures, display hypometabolism (Nestor, Fryer, Smielewski, & Hodges, 2003). In parallel, a recent study has suggested that amyloid deposition begins in key hubs of the DMN, such as the precuneal, medial orbitofrontal and posterior cingulate cortices (Palmqvist et al., 2017). Furthermore, several studies have also demonstrated reduced functional connectivity of the DMN in people with Alzheimer's disease at rest (Gili et al., 2011; Greicius, Srivastava, Reiss, & Menon, 2004; He et al., 2007; Yi et al., 2015; Zhou et al., 2010). From a cognitive perspective, in addition to being implicated in internal mentation processes understood to govern such states of rest (Andrews‐Hanna et al., 2014; Mason et al., 2007), several studies have also shown that it is associated with a variety of memory‐based cognitive tasks (Piccoli et al., 2015; Vatansever, Manktelow, Sahakian, Menon, & Stamatakis, 2016; Vatansever, Menon, Manktelow, Sahakian, & Stamatakis, 2015; Vatansever, Menon, & Stamatakis, 2017).

On the other hand, investigations into the DMN's functional connectivity architecture in people with Down's syndrome have been limited, with only a small number of studies reporting on increased inter‐network connectivity of the DMN in this population (Anderson et al., 2013; Vega, Hohman, Pryweller, Dykens, & Thornton‐Wells, 2015). Despite the demonstrated links to both Alzheimer's disease pathology and the DMN, there has been no investigation into this network's neurodevelopmental alterations in people with Down's syndrome, nor of the potential additive influence of Alzheimer's disease neuropathology.

As such, the present study employed multimodal neuroimaging techniques (fMRI and ^11^C‐PiB PET) with the aim of (a) determining potential functional connectivity alterations of the DMN in people with Down's syndrome, (b) examining the relationship between DMN connectivity and age, IQ and performance on tasks of memory and executive function in people with Down's syndrome and (c) investigating differences in DMN connectivity in people with Down's syndrome with and without fibrillar Aβ accumulation, indicative of Alzheimer's disease neuropathology.

## 2 MATERIALS AND METHODS

### 2.1 Participants

Ethical review and approval of this study were provided by the National Research Ethics Service, East of England Committee. The majority of participants in this study were able to provide informed consent. However, in the minority of cases where participants were not able to consent for themselves, the procedures outlined in the Mental Capacity Act (2005) were followed, and an appropriate consultee was identified who provided permission for the person with Down's syndrome to participate, while the participant provided their assent. A total of 36 participants with Down's syndrome underwent neuropsychological testing, resting state fMRI and ^11^C‐PiB PET. However, one participant was excluded from subsequent analysis due to the presence of ventriculomegaly, which prevented registration to standard templates, and another was excluded due to suspected co‐morbid Parkinson's disease. Therefore, a total of 34 people with Down's syndrome were included (Table 1). A group of 20 age‐matched typically developing control participants underwent resting‐state fMRI only.

**Table 1:** Demographic data for the participant cohort

	Controls	Down's syndrome
Number	20	34
Gender (% female)	45	53
Median age	43.5	43.5
Age range (years)	30–55	30–55

### 2.2 Procedures

#### 2.2.1 Neuropsychological assessment

The CAMDEX‐DS (Ball, 2006) battery was administered to all participants with Down's syndrome. The battery comprises an informant interview (the CAMDEX‐DS) and a neuropsychological examination of the participant (the CAMCOG‐DS).

The diagnosis of dementia was based on findings from the CAMDEX informant interview, conducted with an informant who had known the participant for at least the last 6 months at the time they participated in the study. The informant interview is used to ask, in a structured way, about those areas of function that are known to deteriorate with the development of dementia, for example, memory, general mental functioning, behaviour etc. Where the informant notes a problem in a given domain, they are asked whether or not this deficit in functioning is new or whether it has always been present. The informant is also asked to give examples. Through this approach, a picture of the presence or not of decline across different cognitive and functional domains is obtained. The findings from the CAMDEX informant interview were subsequently discussed with a clinician (SZ or TH) blind to the age and gender of the participant, in order to determine whether or not the participant met the criteria for having developed clinical Alzheimer's disease as outlined in the International Classification of Diseases 10 (ICD). The CAMDEX informant interview has been shown to be valid and reliable as a diagnostic tool (Ball et al., 2004).

Meanwhile, from the CAMCOG‐DS neuropsychological assessment, the memory for new learning subtest (scored out of 21) was used in subsequent analyses, given the propensity for Alzheimer's disease to affect this type of memory. Moreover, the subscale for language (scored out of 27) was also employed in our analyses. In addition, participants completed a modified version of the Tower of London executive function test (Krikorian, Bartok, & Gay, 1994) adapted for people with intellectual disability by Ball and colleagues (Ball, Holland, Treppner, Watson, & Huppert, 2008), scored out of 12. IQ was measured using the raw IQ scores from the Kaufmann Brief Intelligence Test, 2nd edition (KBIT‐II; Kaufman & Kaufman, 2004), since using standard scores would result in many participants scoring at floor (Sinai, Hassiotis, Rantell, & Strydom, 2016).

#### 2.2.2 PET data acquisition

All neuroimaging procedures were carried out at the Wolfson Brain Imaging Centre (WBIC), University of Cambridge, UK. The presence (or absence) of fibrillar Aβ neuropathology in the brains of participants with Down's syndrome was determined using ^11^C‐PiB PET. All ^11^C‐PiB PET scanning and data analysis protocols have been previously described in detail (see Landt et al. (2011) and Annus et al. (2015)). A brief summary of these procedures can be found in the Supporting Information. Based on the outcome of the ^11^C‐PiB PET scan, participants were allocated to PiB‐negative and PiB‐positive groups on the basis of a bimodal distribution in striatal BP_ND_ values, as described in Annus et al. (2015). ^11^C‐PiB PET data were not collected for typically developing controls.

#### 2.2.3 MRI data acquisition

MRI scans were conducted using a 3 T Siemens Verio scanner with a 12‐channel head coil. Participants underwent a T1 weighted magnetisation prepared rapid gradient echo (MPRAGE) scan with the following parameters; TR/TE/TI = 2300/2.98/900 ms, FA = 9°, matrix size 240 × 256, voxel resolution = 1 mm isotropic, receiver bandwidth 240 Hz/pixel, echo spacing 7.1 ms. Parallel acceleration was not enabled. Resting state fMRI data were obtained using a T2* weighted gradient echo, echo planar imaging (EPI) sequence. A total of 298 volumes were acquired in 31 slices (slice thickness 3 mm) with 3 mm isotropic voxels, using the following parameters: TR/TE/2000/30 ms, FA = 78°, FOV = 192 mm × 192 mm × 116 mm, matrix size = 64 × 64. This sequence lasted for 10 min, during which participants were instructed to close their eyes but to stay awake.

### 2.3 Resting‐state fMRI data analysis

Resting‐state fMRI data were preprocessed using the Statistical Parametric Mapping (SPM) v12 software package (http://www.fil.ion.ucl.ac.uk/spm/) based on the v15a MATLAB platform (http://www.mathworks.co.uk/products/matlab/). Functional data were slice time and motion corrected. Structural T1 images were co‐registered to the corresponding mean functional image for each participant, and segmented into three tissue classes (grey matter, white matter and CSF) using the tissue priors from SPM as input, which were normalised to the Montreal Neurological Institute (MNI) 152 space alongside all functional volumes, using the unified‐segmentation method (Ashburner & Friston, 2005). Finally, images were smoothed with an 8 mm FWHM Gaussian kernel.

We anticipated that participants with Down's syndrome may display substantial movement during scanning. Therefore, following recent reports (Ciric et al., 2017), a motion scrubbing procedure was implemented using the artefact detection tools (ART) toolbox, to identify volumes that were motion outliers, while group differences in motion were tested using two measures; the root mean square of frame‐to‐frame percentage change in BOLD signal (DVARS) and mean frame‐wise displacement (FD; Power, Barnes, Snyder, Schlaggar, & Petersen, 2012). The CompCorr method of noise reduction (Behzadi, Restom, Liau, & Liu, 2007) was also employed, whereby five principal components from CSF and white matter noise ROIs were entered into the regression analysis as nuisance variables, alongside the motion outliers identified by the ART procedure. This was done in place of the using the mean global signal as a confounding variable in the GLM, as this can induce spurious negative correlations in resting state analysis (Murphy, Birn, Handwerker, Jones, & Bandettini, 2009), and has recently been demonstrated to impact variance in the analysis associated with group differences (Hahamy et al., 2014).

A seed‐based functional connectivity analysis of the DMN to every other voxel in the brain was performed using the *Conn* functional connectivity toolbox (Susan Whitfield‐Gabrieli & Nieto‐Castanon, 2012), with a spherical medial prefrontal cortex seed (10 mm in diameter) centred on the following MNI coordinates: [−1, 47, −4]. The medial prefrontal cortex is a hub region of the DMN that has been previously used in seed‐based investigations of DMN connectivity (Keller et al., 2015). In the first level analysis, Pearson correlation coefficients between the residual BOLD time‐series of the seed region and that of every other voxel in the brain were calculated using the general linear model (GLM). These correlation coefficients were subsequently Z‐transformed, yielding a correlation map for each participant. For comparison, an analysis was also carried out using a seed located in the posterior parietal cortex (PPC; see Supporting Information).

Second level analysis took the form of within‐group *t*‐tests and between groups *t*‐tests to identify differences in DMN connectivity between the typically developing control group and the whole Down's syndrome cohort (referred to as Down's Syndrome [all] in the following sections). Additional between‐groups tests were conducted to characterise the separate contributions of Down's syndrome and the presence of fibrillar Aβ neuropathology to alterations in DMN connectivity. To determine differences in DMN connectivity that were principally due to the presence of Down's syndrome, the PiB‐negative Down's syndrome group were compared to the control group. Meanwhile, the effects of the presence of fibrillar Aβ on DMN connectivity were assessed by comparing the PiB‐positive and PiB‐negative Down's syndrome groups to each other. As a supplementary analysis, the compound effect of having both Down's syndrome and fibrillar Aβ neuropathology on DMN connectivity was determined by comparing the PiB‐positive Down's syndrome group and the control group. All reported results are cluster corrected using the family‐wise error (FWE) detection technique at the *p* < .05 level of significance (uncorrected at the voxel level, *p* < .001).

The relationships between DMN connectivity and various demographic and cognitive measures were investigated in the Down's syndrome (all) group using a whole‐brain GLM approach. To do this, connectivity maps for the Down's syndrome (all) group were entered into a one‐sample *t*‐test with each metric (age, raw IQ score, memory for new learning, language and Tower of London executive function task score). Due to variations in the severity of intellectual disability and/or cognitive decline among participants with Down's syndrome, some participants were unable to complete all tests. Therefore, while the number of participants included varied from test to test, the minimum number of participants included in each analysis was 30. All measures were mean centred, and both positive and negative contrasts were specified to investigate connections that were both positively and negatively associated with task performance. To determine the strength of the correlation, connectivity values from the peak voxel of each significant cluster were extracted and entered into a Spearman correlation analysis alongside the mean centred neuropsychological test scores.

### 2.4 Statistical analysis

Statistical tests were carried out using the IBM SPSS statistics package, Version 22 (https://www.ibm.com/products/spss-statistics). Due to the non‐normal distribution of a number of variables in the dataset, nonparametric statistics were employed. Differences in age between the typically developing control and Down's syndrome (all) groups were investigated using the Mann–Whitney *U* test. This test was also used to investigate differences between the PiB‐negative Down's syndrome and PiB‐positive Down's syndrome groups in raw IQ score, memory for new learning, language and performance on the Tower of London executive function task.

Meanwhile, differences in gender distribution across three groups (i.e. typically developing controls, PiB‐negative Down's syndrome and PiB‐positive Down's syndrome) were investigated using the chi‐square test of association, while statistical differences in age across these three groups were investigated using the Kruskal–Wallis H test, followed by *post hoc* tests using Dunn's procedure. Finally, the relationship between performance on tests of IQ/cognitive function and default mode connectivity in the Down's syndrome (all) group was investigated using a whole‐brain approach in SPM.

## 3 RESULTS

There were no statistically significant differences between the typically developing control and Down's syndrome (all) groups in terms of age (*U* = −0.224, *p* = .823) or gender distribution (χ^2^ = 0.318, *p* = .573). As expected, the two groups differed significantly in terms of motion during scanning, with the Down's syndrome (all) group showing a greater number of volumes affected by motion (see Table S1).

### 3.1 Default mode network in Down's syndrome

Our first aim was to determine the differences between typically developing controls and people with Down's syndrome irrespective of Aβ pathology. As such, the initial analyses investigated DMN connectivity within and between the typically developing and Down's syndrome (all) groups.

**Figure 1:**
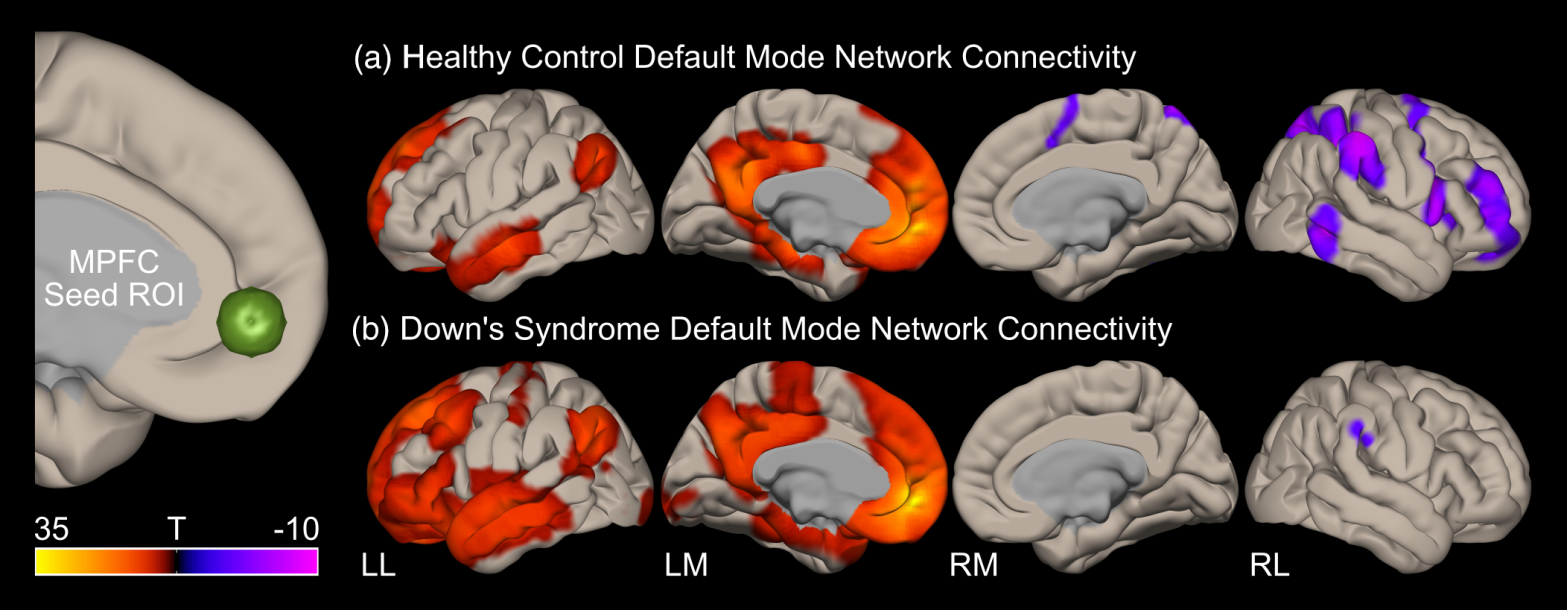
Within group default mode network connectivity in (a) typically developing age‐matched controls and (b) all participants with Down's syndrome. The statistical maps (T‐scores) represent positive correlation (red) and anti‐correlation (blue) of the medial prefrontal cortex (mPFC) seed region (connectivity with subcortical structures and cerebellum is not shown) [Color figure can be viewed at http://wileyonlinelibrary.com]

The within‐group profiles of DMN connectivity (using the medial prefrontal cortex seed) in these two groups can be seen in Figure 1. While this analysis yielded the expected pattern of DMN connectivity for the control group (Andrews‐Hanna, 2012), the Down's syndrome (all) group did not display a typical profile of DMN connectivity. Rather, functional connectivity of the DMN seed to the rest of the brain was far more extensive in the Down's syndrome (all) group. Furthermore, although the control group displayed a similar pattern of anti‐correlation with the DMN to that seen in previous studies (Fox et al., 2005), there was a strikingly different pattern of anti‐correlation with the DMN in the Down's syndrome (all) group, in that almost no anti‐correlation with other cortical regions was observed.

**Figure 2:**
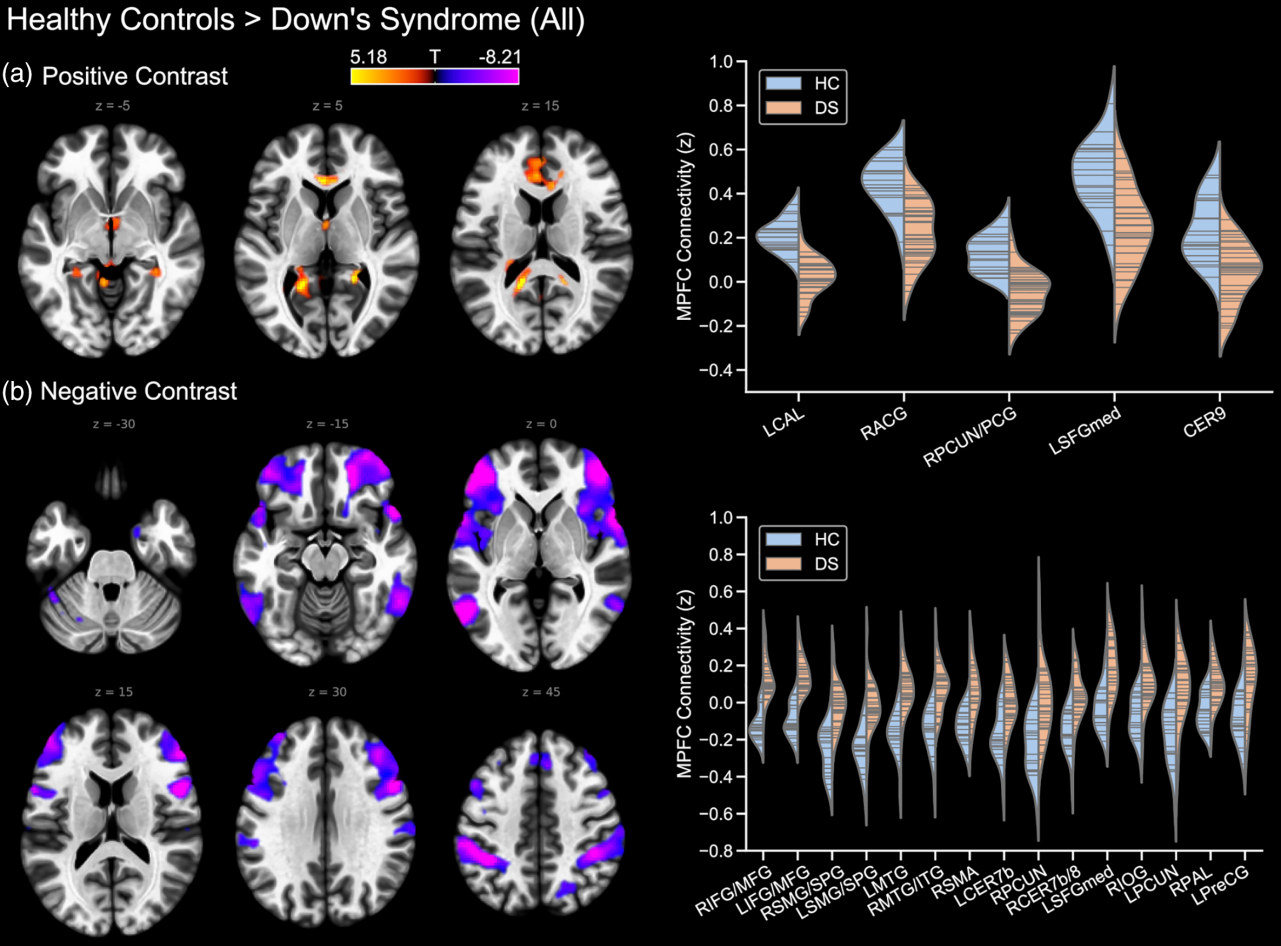
Differences in default mode network connectivity (mPFC seed) between the typically developing control and Down's syndrome groups, showing (a) controls > Down's syndrome (all), and (b) Down's syndrome (all) > typically developing controls. Where prefixed to the abbreviation of a brain region, L and R indicate the left and right hemisphere, respectively. ACG, anterior cingulate gyrus; CAL, calcarine cortex; CER9, region 9 of the cerebellum; CER7b, region 7 of the cerebellum; IFG, inferior frontal gyrus; IOG, inferior occipital gyrus; ITG, inferior temporal gyrus; MFG, inferior/middle frontal gyrus; MTG, middle temporal gyrus; PAL, pallidum; PCG, posterior cingulate gyrus; PCUN, precuneus; PreCG, precentral gyrus; SFGmed, medial superior frontal gyrus; SMA, supplementary motor area; SMG, supramarginal gyrus; SPG, superior parietal gyrus [Color figure can be viewed at http://wileyonlinelibrary.com]

To quantify the differences seen in the within groups analysis, paired comparisons were conducted between the typically developing control group and the Down's syndrome (all) cohort. The contrast of controls > Down's syndrome (all) showed that the latter group had reduced (i.e. weaker) connectivity between the DMN (medial prefrontal cortex seed) in the following regions: the right precuneus/posterior cingulate, the left calcarine and the right anterior cingulate cortices, the medial superior frontal gyrus, as well as region 9 of the cerebellum (Figure 2a). Conversely, the contrast of Down's syndrome (all) > controls revealed positive connectivity in the Down's syndrome group to regions that were anti‐correlated with the DMN in the control group (Figure 2b). Greater positive DMN connectivity in the Down's syndrome (all) group was seen in the pars orbitalis and triangularis of the middle frontal gyrus, the middle temporal gyrus, the supplementary motor area bilaterally, the left precentral gyrus, the right inferior occipital cortex and the right putamen. However, other regions were strongly anti‐correlated in the control group but were only weakly correlated with the DMN in the Down's syndrome (all) group, including regions of parietal lobes bilaterally, region 7b of the cerebellum bilaterally and the right precuneus. Finally, there was strong connectivity of the DMN to the left medial superior frontal gyrus in the Down's syndrome (all) group that was not present in the control group.

### 3.2 Default mode alterations in the absence/presence of fibrillar Aβ neuropathology

It has been well established that Alzheimer's disease affects the connectivity of the DMN (Buckner et al., 2005; Greicius et al., 2004; Whitfield‐Gabrieli & Ford, 2012). Therefore, to determine the effects of the accumulation of Alzheimer's disease neuropathology, specifically fibrillar Aβ, on DMN connectivity in people with Down's syndrome, the Down's syndrome (all) group was divided into those negative and positive for cerebral fibrillar Aβ, as determined by ^11^C‐PiB PET. A Kruskal–Wallis H test indicated a statistically significant effect of age across the three groups (χ^2^ = 10.074, *p* = .006), with *post hoc* tests using Dunn's procedure and the Bonferroni correction for multiple comparisons indicating that this was driven by a difference between the PiB‐negative and PiB‐positive Down's syndrome groups (with a median age of 42 and 48, respectively, *p* = .005). Further demographic and neuropsychological data for the PiB‐negative and PiB‐positive Down's syndrome groups are shown in Table 2.

**Table 2:** Demographic data and neuropsychological test results for PiB‐negative and PiB‐positive Down's syndrome groups

	TD controls	PiB –ve	PiB +ve	Effect size	*p*
Number	20	19	15	–	–
Gender (% female)	45	57	46.67	χ^2^ = 0.740	.691
Age	43.5	41.0	48.0**	χ^2^ = 10.074	.006
Age range	30–55	30–48	39–55	–	–
DVARS	25.5	31.87*	37.85*	χ^2^ = 26.467	<.0005
FD	0.17	0.34*	0.39*	χ^2^ = 24.512	<.0005
Raw IQ	–	56	60	*U* = 107.5	.482
Tower of London	–	9	6.5	*U* = 64.5	.018
New learning	–	14	9	*U* = 70.5	.011
Language	–	15	15	*U* = 97	.111
Number with dementia	–	3	5	–	–

FD, Frame‐wise displacement; IQ, intelligence quotient; PiB –ve, PiB‐negative; PiB +ve, PiB‐positive; D, typically developing.

* Significantly different from controls at the level of *p* < .0005

** Significantly different from PiB‐negative Down's syndrome at the level of *p* < .005.

**Figure 3:**
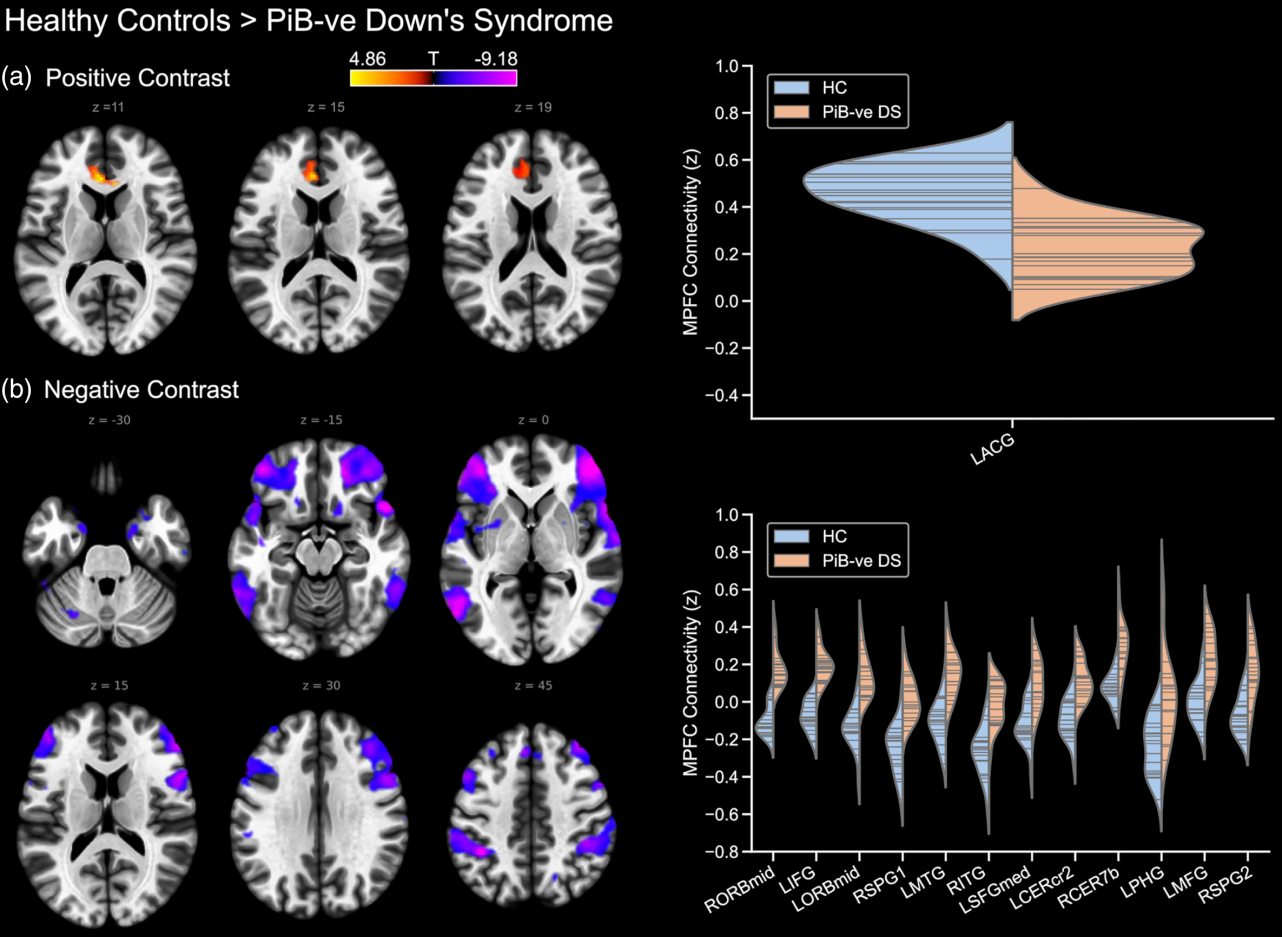
Differences in default mode network connectivity between typically developing controls and PiB‐negative participants with Down's syndrome, showing (a) controls > PiB −ve Down's syndrome, and (b) PiB −ve Down's syndrome > controls. PiB −ve, PiB‐negative; where prefixed to the abbreviation of a brain region, L and R indicate the left and right hemisphere, respectively. Furthermore, a suffix of 1 or 2 to an abbreviation serves to differentiate different significant clusters located within a single brain region. ACG, anterior cingulate gyrus; CERcr2, cerebellum crus 2; CER7b, region 7b of the cerebellum; ITG, inferior temporal gyrus; MFG, middle frontal gyrus; MTG, middle temporal gyrus; ORBmid, middle orbitofrontal gyrus; PHG, parahippocampal gyrus; SPG, superior parietal gyrus; SFGmed, medial superior frontal gyrus [Color figure can be viewed at http://wileyonlinelibrary.com]

To investigate the effects of Down's syndrome (i.e. in the absence of fibrillar Aβ neuropathology) on DMN connectivity, the PiB‐negative group were compared to the typically developing control group. The contrast of controls > PiB‐negative Down's syndrome showed the latter group had reduced connectivity between the DMN (medial prefrontal cortex seed) and the left anterior cingulate (Figure 3). The reverse contrast, however, revealed greater positive DMN connectivity in the Down's syndrome group in a distribution of regions that were anti‐correlated with the DMN in the control group, and highly similar to that seen in the comparison between the controls and the whole Down's syndrome group (all) described above.

**Figure 4:**
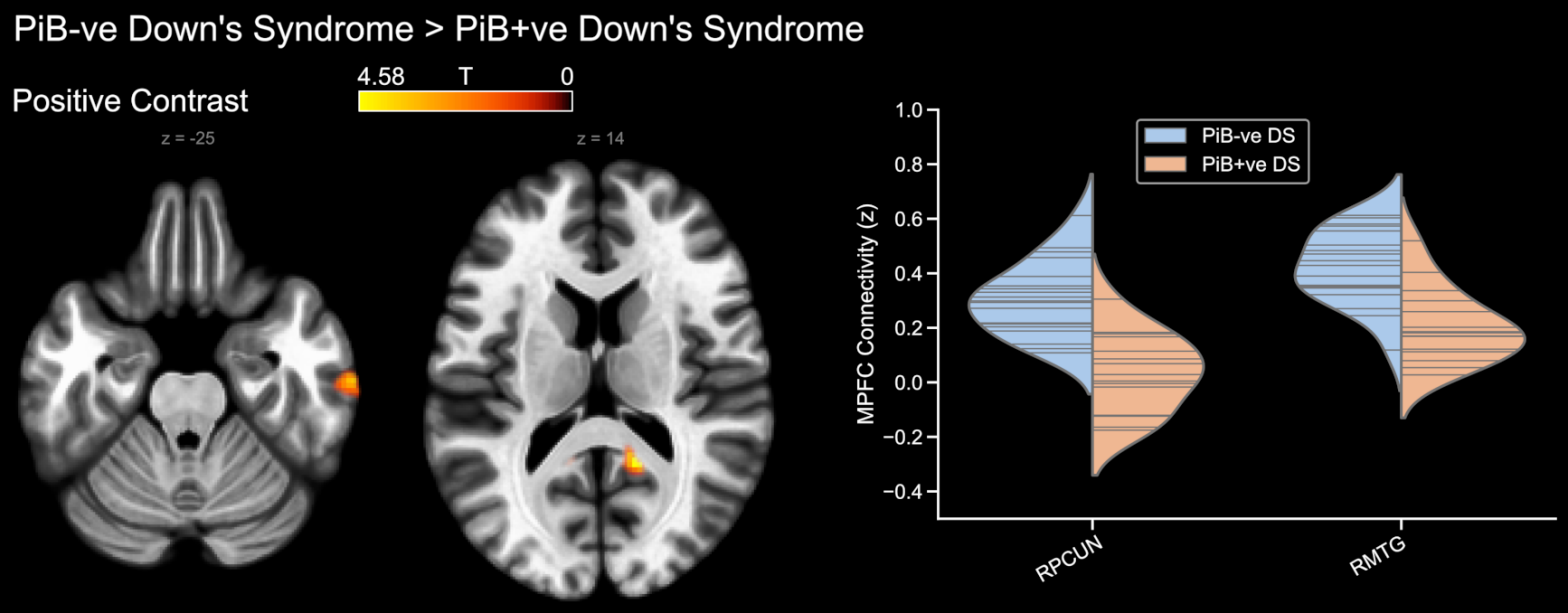
Differences in default mode network connectivity between the PiB‐negative and PiB‐positive Down's syndrome groups (PiB‐negative > PiB‐positive). The reverse contrast is not shown. PiB –ve, PiB‐negative; PiB +ve, PiB‐positive; RMTG, right middle temporal gyrus; RPCUN, right precuneus [Color figure can be viewed at http://wileyonlinelibrary.com]

Meanwhile, to determine the effect of Aβ neuropathology (i.e. exclusive of the effects of Down's syndrome), the PiB‐positive Down's syndrome group were compared to the PiB‐negative Down's syndrome group. The contrast of PiB‐negative Down's syndrome > PiB‐positive Down's syndrome showed that the PiB‐positive group had reduced DMN connectivity to the precuneus and posterior cingulate cortex, as well as to a region of the right middle temporal gyrus (Figure 4). However, the reverse contrast did not reveal any statistically significant differences.

**Figure 5:**
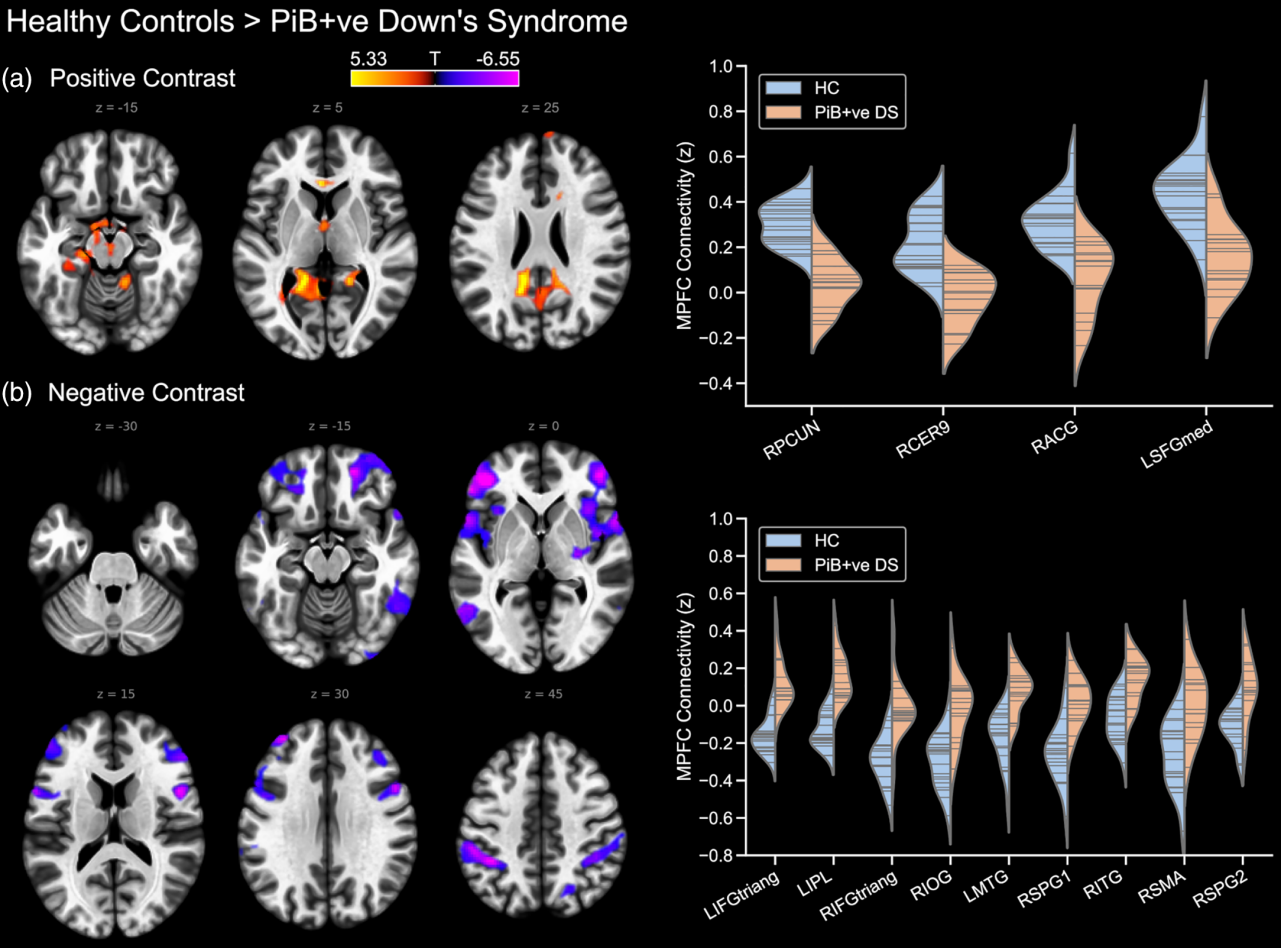
Default mode network connectivity in the PiB‐positive Down's syndrome group compared to the typically developing control group, showing (a) controls > PiB‐positive Down's syndrome, and (b) PiB‐negative Down's syndrome > controls. PiB +ve, PiB‐positive; prefixed to the abbreviation of a brain region, L and R indicate the left and right hemisphere, respectively. Furthermore, a suffix of 1 or 2 to an abbreviation serves to differentiate different significant clusters located within a single brain region. ACG, anterior cingulate gyrus; CER9, region 9 of the cerebellum; IFGtriang, inferior frontal gyrus pars triangularis; IOG, inferior occipital gyrus; IPL, inferior parietal lobule; ITG, inferior temporal gyrus; MTG, middle temporal gyrus; PCUN, precuneus; SFGmed, medial superior frontal gyrus; SMA, supplementary motor area; SPG, superior parietal gyrus [Color figure can be viewed at http://wileyonlinelibrary.com]

Finally, a comparison of the typically developing control and PiB‐positive Down's syndrome groups was conducted to investigate the compound effects of Down's syndrome and Aβ neuropathology on DMN connectivity. The PiB‐positive Down's syndrome group had reduced DMN connectivity to the precuneus and posterior cingulate cortex, the anterior cingulate, region 9 of the right cerebellum and the left medial superior frontal gyrus (Figure 5), as well as to regions of the left parahippocampal gyrus, left amygdala and the hippocampus and thalamus bilaterally. Meanwhile, the reverse contrast showed that the PiB‐positive Down's syndrome group had positive DMN connections to many regions which were anti‐correlated with the DMN in the control group, including the inferior and superior parietal lobules, the middle frontal gyri and the right inferior temporal gyrus.

### 3.3 Association of default mode alterations and cognitive performance

**Figure 6:**
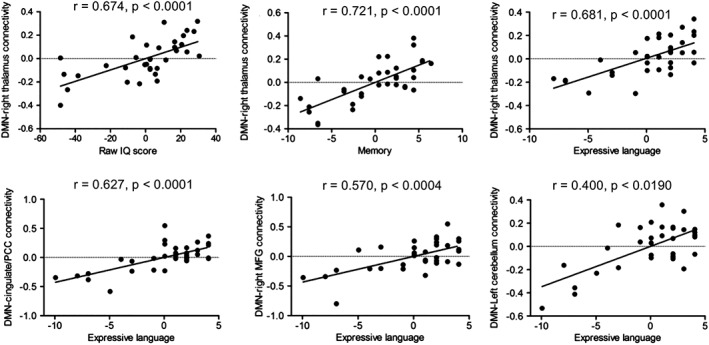
Correlations between IQ, memory and language and default mode network (DMN) connectivity in the Down's syndrome (all) group. MFG, middle frontal gyrus; PPC, posterior cingulate cortex

As the Down's syndrome (all) group also represented various degrees of risk for cognitive decline and Alzheimer's disease, the relationship of DMN connectivity to IQ and cognition were investigated in this group (Figure 6) using a whole‐brain approach in SPM. There was a strong positive correlation between DMN connectivity to the right thalamus and raw IQ score (*r* = .674, *p* < .0001), memory for recently learned information (*r* = .721, *p* < .0001) and language (*r* = .681, *p* < .0001), indicating that better scores on tests measuring these faculties were correlated with stronger connectivity between the DMN and the right thalamus. Language score also correlated positively with DMN connectivity to the left middle cingulate and posterior cingulate cortex (*r* = .627, *p* < .0001), the right middle frontal gyrus (*r* = .570, *p* < .0004) and regions 4 and 5 of the left cerebellum (*r* = .400, *p* < .0190). There was no association between DMN connectivity and scores on the Tower of London test of executive function.

## 4 DISCUSSION

Overall, the results of this study highlight widespread alterations in the connectivity of the DMN in people with Down's syndrome. Pairwise comparisons between the Down's syndrome (all) group and the typically developing controls revealed a pattern of weaker connectivity in the Down's syndrome group between the DMN seed and a number of regions, including the posterior cingulate/precuneus, as well as the anterior cingulate, the left calcarine cortex, the medial superior frontal gyrus and part of the cerebellum. Furthermore, dividing the Down's syndrome population into those negative and positive for fibrillar Aβ neuropathology as determined by ^11^C‐PiB PET indicated that reductions in the strength of DMN connectivity to frontal brain regions may be largely associated with Down's syndrome, while reductions in DMN connectivity to posterior brain regions are linked to the presence of Alzheimer's disease neuropathology.

Notably, this last result suggests that the findings of this study may have a wider significance (i.e. extending to other Alzheimer's disease patient populations) as the pattern of reduced connectivity in the presence of Alzheimer's disease pathology in the PiB‐positive Down's syndrome group was reminiscent of that which has been shown to occur in sporadic and familial Alzheimer's disease (Chhatwal et al., 2013; Gili et al., 2011; Greicius et al., 2004; He et al., 2007; Yi et al., 2015; Zhou et al., 2010). That is, the alterations in DMN connectivity seen in this study encompassed regions including the posterior cingulate cortex and precuneus, a part of the brain that also shows altered metabolic function in people with Alzheimer's disease and is a key site of Aβ deposition (Buckner et al., 2005; Buckner et al., 2009). As such, this study serves to highlight the similarities in the effects of Alzheimer's disease on the brains of people with Down's syndrome and typically developing controls, indicating the utility of involving people with Down’ syndrome in research, since the development of Alzheimer's disease can more readily be predicted in this patient population, but the results may be, in large part, generalisable.

Alterations in the DMN connectivity of the posterior cingulate cortex and precuneus have also been seen in an asymptomatic population at high risk for Alzheimer's disease compared to asymptomatic individuals with no increased risk (Chhatwal et al., 2013). This is particularly interesting given the largely preclinical nature of the cohort involved in the present study, and taken together these findings may corroborate the notion that disrupted functional connectivity of the DMN is an early biomarker of Alzheimer's disease neuropathology. However, this finding was not replicated in a subsequent study that included slightly younger asymptomatic populations at risk of sporadic Alzheimer's disease due to the presence of the risk allele APOE ε4 (Thomas et al., 2014). Yet it must be noted that in the case of the study of a familial autosomal dominant Alzheimer's disease cohort by Chhatwal et al. (2013), and in the case of people with Down's syndrome as in the present study, the onset of Alzheimer's disease neuropathology is a near certainty provided that the required mutation or additional copy of APP is present, and therefore may represent a qualitatively different population to those with other risk factors such as APOE ε4 alleles, which are not determinant but greatly increase risk.

Thus, the similarities between the present findings and those of other studies involving different clinical populations further highlight the candidacy of people with Down's syndrome for future Alzheimer's disease research. Moreover, given that the development of neuropathology is more predictable in this cohort as compared to sporadic Alzheimer's disease, the design of clinical trials for preventative treatments may be less costly and more efficient. However, before any such future studies can be carried out, it is necessary to consider the problem inherent in all Alzheimer's disease research, but which is particularly relevant when conducting studies involving people with Down's syndrome; that is, the problem of age. Given the robust link between age and amyloid deposition in people with Down's syndrome, effects attributed to the presence of amyloid cannot readily be disentangled from effects due to age. In the present study, age was not entered as a covariate into the present analysis for this very specific reason, since by removing the effect of age from any analysis, one is certain to also remove a great deal of variance that may be due to the presence of Aβ neuropathology.

Furthermore, while the findings of the present study bring to light important new information regarding DMN connectivity in people with Down's syndrome in relation to the development of amyloid pathology, this study also has implications for future investigations of DMN connectivity in people with Down's syndrome from a developmental perspective. Our results demonstrated a highly atypical DMN in people with Down's syndrome, characterised by widespread positive connectivity (i.e. hyper‐connectivity) coupled with a striking reduction in anti‐correlation. This effect was noticeable in the within‐group analysis of DMN connectivity for the Down's syndrome (all) group but was also emphasised by the between‐group analysis contrasting the Down's syndrome (all) group and typically developing controls. This latter comparison showed positive connectivity of the DMN in this Down's syndrome group to regions that were negatively correlated with the DMN in the control group. Notably, once the Down's syndrome (all) group was divided into those negative and positive for the presence of fibrillar Aβ neuropathology, the hyper‐connectivity effect was seen most prominently in the PiB‐negative Down's syndrome group, indicating that it may be the de‐facto, premorbid (i.e. pre‐Alzheimer's disease) state of the DMN in this population.

This pattern of DMN hyper‐connectivity is similar to findings reported from previous fMRI investigations of brain connectivity involving people with Down's syndrome (Anderson et al., 2013; Vega et al., 2015). These studies found increased inter‐network connectivity in people with Down's syndrome compared to controls, including greater positive connectivity between the default mode network and numerous other large‐scale functional brain networks. Together, these studies suggest that the organisation of the brain into segregated networks (Fox et al., 2005) is highly disrupted in people with Down's syndrome.

Whether and how this hyper‐connectivity of the DMN is related to developmental cognitive dysfunction and intellectual disability seen in people with Down's syndrome is an important question and one that should be taken up by future research. The present study may act as a catalyst for such studies, having found associations between performance on tests of IQ, memory for new learning, language and DMN connectivity to various regions, most prominently the right thalamus.

As noted above, the hyper‐connectivity of the DMN in people with DS revealed in this study was coupled with a lack of anti‐correlation with the DMN. In the control group, the presence of a network that is anti‐correlated with the DMN was evident from the within‐group analysis and encompassed many of the regions of the “task‐positive network,” described by Fox et al. (2005). Notably, the exact physiological nature of the anti‐correlation between brain networks is still under debate (Chai, Castanon, Ongur, & Whitfield‐Gabrieli, 2012; Murphy et al., 2009), with recent publications highlighting their potential biological basis (Fox, Zhang, Snyder, & Raichle, 2009; Keller et al., 2015; Spreng, Stevens, Viviano, & Schacter, 2016), and indicating that they may provide a level of segregation between two major brain systems for healthy cognitive processing (Gao & Lin, 2012; Uddin, Kelly, Biswal, Castellanos, & Milham, 2009; Vatansever et al., 2017).

The notion that anti‐correlation between functionally distinct networks is necessary for normal brain function is somewhat intuitive, as it neatly lends itself to a mechanism by which distinct functional networks can be organised and segregated within the brain (Fox et al., 2005). Furthermore, it is an idea that is given some weight by consistent findings of reductions in anti‐correlation with the DMN in other brain disorders, including Alzheimer's disease (Wang et al., 2007), attention‐deficit/hyperactivity disorder (Castellanos et al., 2008), autism (Anderson et al., 2011), schizophrenia (Whitfield‐Gabrieli et al., 2009) and behavioural frontotemporal dementia (Hafkemeijer et al., 2015). Thus, the hyper‐connectivity of the DMN in people with Down's syndrome (although largely weaker than in controls in areas such as the anterior cingulate) and the (almost) complete absence of anti‐correlation may be a large contributing factor to the disorganisation of the DMN in this group of people.

Finally, it is important to consider the differences in brain morphology between people with Down's syndrome and typically developing controls, and its possible impact on functional connectivity estimates.

A number of volumetric MRI studies primarily employing ROI‐based approaches (please see Annus et al., 2017 for a summary) have identified reduced overall grey matter volumes in people with Down's syndrome relative to controls (Beacher et al., 2010; Pearlson et al., 1998; White, Alkire, & Haier, 2003), while specific reductions in volume have also been reported in the frontal lobes, the hippocampus and the cerebellum (Aylward et al., 1999; Beacher et al., 2010; Koran et al., 2014; Pearlson et al., 1998; White et al., 2003). Meanwhile, increases in grey matter volume have been noted in the parahippocampal gyrus and in the parietal and occipital cortices (Beacher et al., 2010; White et al., 2003).

Notably, however, where cortical thickness in Down's syndrome is concerned, Annus et al. (2017) have demonstrated that those without fibrillar amyloid (as determined using 11C‐PiB PET) show a thickening of the cortex relative to typically developing controls in the lateral and medial frontal, parietal and occipital cortices and in the region of the precuneus/posterior cingulate cortex. These findings have been corroborated to some degree in a recent study by Levman et al. (2019) of infants, children and young adults with Down's syndrome, which found thicker cortex in the participants with Down's syndrome in frontal regions, among others, including Brodmann's area, the medial and lateral orbitofrontal cortex and the anterior cingulate.

Meanwhile, markedly reduced cortical thickness was seen by Annus et al. (2017) in PiB‐positive participants with Down's syndrome (relative to the PiB‐negative group) in posterior regions including the precuneus and posterior cingulate, and the parietal, occipital and posterior temporal lobes, indicating a pattern of thinning in this group remarkably similar to that seen in sporadic Alzheimer's disease (Annus et al., 2017).

Due to concerns regarding these morphological differences between the brains of people with Down's syndrome and the typically developing population, the present study included a supplementary analysis using a seed centred on the posterior parietal cortex (PPC) [MNI: −5, −51, 39], to determine whether the choice of an mPFC seed may be unduly influencing the results. While the results of this analysis (displayed in Figure S1) show a slightly different pattern of default mode connectivity differences in the Down's syndrome group relative to controls, with more posterior dominance, the overall direction of results remains the same, with substantial regions of reduced anti‐correlation to the DMN being seen in participants with Down's syndrome. Moreover, when comparing the PiB‐negative and PiB‐positive Down's syndrome groups using the PPC seed, a near identical pattern of results to that obtained using the mPFC seed is observed, in that the PiB‐positive group showed a specific reduction in positive connectivity of the PPC seed to the posterior cingulate cortex. As such, the results of this additional analysis indicate that the differences we have observed in this study can be generalised to the whole DMN and are not likely to be the consequence of differences in brain morphology or the selection of a specific seed region. Furthermore, the findings of Annus et al. (2017) regarding cortical thinning in PiB‐positive participants with Down's syndrome in the regions encompassing the PPC seed indicate that the use of an mPFC seed for comparing DMN connectivity between these two groups is the more appropriate choice.

In summary, the present study has demonstrated widespread disruption of the DMN in people with Down's syndrome, which is further altered in the presence of Aβ neuropathology in a pattern that is reminiscent of that seen in other populations with Alzheimer's disease. The significance of these findings may be further highlighted given the largely preclinical nature of our cohort, indicating that functional connectivity of the DMN may be useful in future studies as an early biomarker of altered neuronal function due to Alzheimer's disease.

5

## Supporting information


**Appendix S1:** Supporting information

**Figure S1** Between groups differences in default mode network connectivity based on a medial prefrontal cortex (mPFC) seed is compared to group differences in default mode connectivity based on a posterior parietal cortex (PPC) seed. The location of the seed region in each analysis is shown in green. HC, healthy control; DS, Down's syndrome; LL, left lateral; LM, left medial; PiB −ve, PiB‐negative; PiB +ve, PiB‐positive; RL, right lateral; RM, right medial; ROI, region of interest.

## Data Availability

Raw data were generated at the Wolfson Brain Imaging Centre, University of Cambridge, UK. Derived data supporting the findings of the study are available from the corresponding author on request.
