# Supplementary material for: Differential effects of Down's syndrome and Alzheimer's neuropathology on default mode connectivity
Source: Hum Brain Mapp. 2019 Jul 26;40(15):4551–63. doi: 10.1002/hbm.24720 (PMC6865660; doi:10.1002/hbm.24720)
Supplement: Supplementary file 1 — Appendix S1: Supporting information [file HBM-40-4551-s002.docx]

**Supplementary Material:** Differential effects of Down’s syndrome and Alzheimer’s neuropathology on default mode connectivity

**Authors:** Liam R. Wilson^1^, Deniz Vatansever^2^, Tiina Annus^1^, Guy B. Williams^3^, Young T. Hong^3^, Tim D. Fryer^3^, Peter J. Nestor,^4^ Anthony J. Holland^1^ and Shahid H. Zaman^1^*

**Affiliations:**

1. Cambridge Intellectual and Developmental Disabilities Research Group, Department of Psychiatry, University of Cambridge, Cambridge, UK.
2. Institute of Science and Technology for Brain-inspired Intelligence, Fudan University, Shanghai, PR China.
3. The Wolfson Brain Imaging Centre, Department of Clinical Neurosciences, University of Cambridge, Cambridge, UK.
4. Queensland Brain Institute, University of Queensland, Brisbane, Australia.

***Corresponding author:**

Cambridge Intellectual and Developmental Disabilities Research Group

Douglas House, University of Cambridge

18b Trumpington Road

Cambridge, CB2 8AH

United Kingdom

**Email:** [shz10@medschl.cam.ac.uk](mailto:shz10@medschl.cam.ac.uk).

Table of Contents

[Supplementary Methods 3](#_Toc11181021)

[Supplementary Results 6](#_Toc11181023)

# Supplementary Methods

**Analysis of PET Data**

The presence (or absence) of fibrillar Aβ neuropathology in the brains of participants with Down’s syndrome was determined using ^11^C-PiB PET. All ^11^C-PiB PET scanning and data analysis protocols have been previously described in detail (see Landt et al., 2011; Annus et al., 2015). Briefly, dynamic ^11^C-PiB PET scans were performed on a GE-Advance scanner at the Wolfson Brain Imaging Centre (WBIC), University of Cambridge, UK. Dynamic scans were preceded by a ^68^Germanium transmission scan to correct for photon attenuation. ^11^C-PiB was produced with high radiochemical purity (> 95%) and specific activity (150 GBq/µM) according to good manufacturing practices in an on-site radiopharmaceutical laboratory. Following a bolus injection of 550 MBq of ^11^C-PiB, participants underwent a 90-minute dynamic scan. Regional BP_ND_ values were extracted through registration of each participant’s mean emission data to a manually improved Brodmann atlas. Subcortical structures were segmented using FSL FIRST ([Patenaude et al., 2011](#_ENREF_27)). Participants were allocated to PiB negative and PiB positive groups on the basis of a bimodal distribution in striatal BP_ND_ values, as described in [Annus et al. (2015](#_ENREF_3)). ^11^C-PiB PET data were not collected for typically developing controls.

**Motion Scrubbing and Analysis of Head Motion**

A motion scrubbing procedure was implemented using the artefact detection tools (ART) toolbox, to identify volumes that were motion outliers. Motion outliers were defined as volumes in which the global signal changed by 3 standard deviations, in which translations exceeded 2mm, or rotation exceeded 0.02 radians. As part of the noise reduction procedure, these motion outliers were entered into a regression analysis as nuisance variables, along with the six motion parameters (three translations and three rotations) and their first order derivatives computed during realignment. The CompCorr method of noise reduction was also employed ([Behzadi et al., 2007](#_ENREF_5)), whereby five principal components from CSF and white matter noise ROIs were additionally entered as nuisance variables. This was done in place of the using the mean global signal as a confounding variable in the GLM, since this can induce spurious negative correlations in resting state analysis ([Murphy et al., 2009](#_ENREF_26)).

In addition to the above-outlined motion scrubbing procedure, we compared levels of head motion between the typically developing control group and the Down’s syndrome (all) group using the root mean square of frame-to-frame percentage change in BOLD signal (DVARS, Power et al., 2012), as well as the mean frame-wise displacement (FD) (Power et al., 2012). These metrics, were derived from realigned data separately from the motion scrubbing procedure implemented with ART, using the fsl_motion_outliers function from FSL (<https://fsl.fmrib.ox.ac.uk/fsl/fslwiki/FSL>). Voxels included in the calculation of DVARS and FD were determined using a mask of each individual’s mean functional image.

DVARS and FD data for the Down’s syndrome (all) and typically developing control groups are displayed in Table S1. Note that median data are displayed and that group differences were tested using the non-parametric Mann-Whitney U test as the data were not normally distributed. Statistical testing was carried out using IBM SPSS statistics package, Version 22 (<https://www.ibm.com/products/spss-statistics>).

| Table S1: Median DVARS and FD | | | | |
| --- | --- | --- | --- | --- |
|  | **Typically developing controls** | **Down’s syndrome (all)** | **Effect size** | **Significance (p)** |
| DVARS | 27.9 | 38.9 | *U* = 642 | < 0.0005 |
| FD | 0.17 | 0.37 | *U* = 1204 | < 0.0005 |

**Posterior Parietal Cortex (PPC Connectivity) Analysis**

As a supplementary analysis to the investigation of medial prefrontal cortex (mPFC) connectivity, the functional connectivity of another default mode network hub, namely the posterior parietal cortex (PPC) was also investigated. The methodology for carrying out this analysis was identical to that employed in determining the connectivity of the mPFC, with the exception that the seed region was focused on the following MNI co-ordinates: [-5, -51, 39] As such, the first level analysis involved calculating Pearson correlation coefficients between the residual BOLD time-series of the seed region (PPC) and that of every other voxel in the brain using the general linear model (GLM). The resulting correlation coefficients were subsequently Z-transformed to yield a correlation map for each participant. Between-group differences in PPC connectivity were also investigated in same way as during the mPFC second level analysis. That is, differences in connectivity to the PPC were initially investigated between typically developing controls and the Down’s syndrome (all) groups by means of two between groups t-tests. Subsequently, additional between groups t-tests were carried out to characterise the separate contributions of Down’s syndrome and the presence of fibrillar Aβ neuropathology to alterations in DMN connectivity (as determined based on the PPC seed). To investigate differences that could be primarily attributed to the presence of Down’s syndrome, the PiB negative Down’s syndrome groups were compared to the control group. Following this, the effects of the presence of fibrillar Aβ on DMN (PPC) connectivity were determined by comparing the PiB positive and PiB negative Down’s syndrome groups. Finally, the compound effect of having both Down’s syndrome and fibrillar Aβ neuropathology was determined by a comparison of the PiB positive Down’s syndrome group and the control group.

# Supplementary Results

The results of the between-groups t-tests carried out using the PPC seed [-5, -51, 39] are displayed in Supplementary Fig. S1, alongside the results obtained using the mPFC seed.


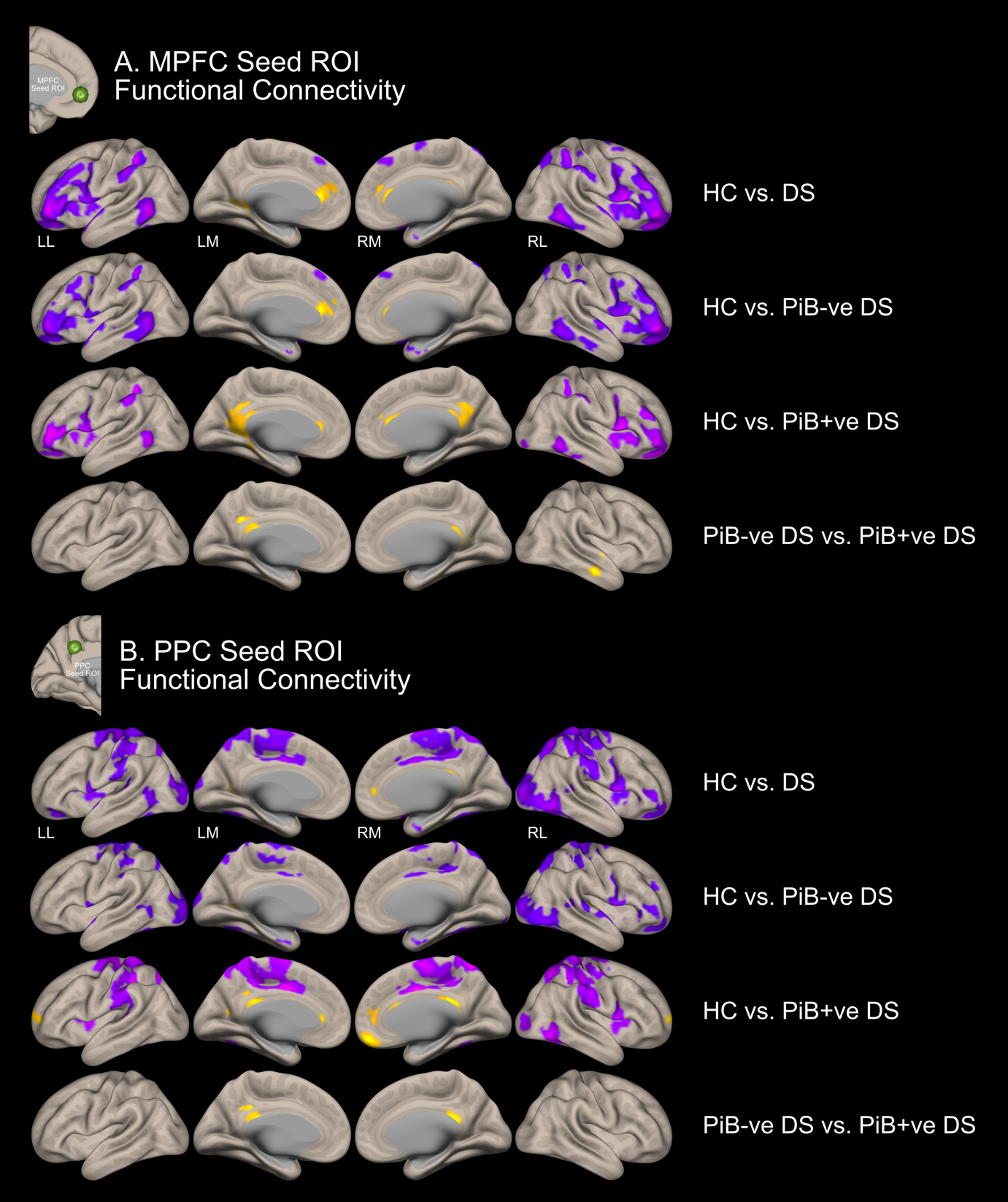


# Supplementary Figure S1. Between groups differences in default mode network connectivity based on a medial prefrontal cortex (mPFC) seed is compared to group differences in default mode connectivity based on a posterior parietal cortex (PPC) seed. The location of the seed region in each analysis is shown in green. Abbreviations: HC – healthy control; DS – Down’s syndrome; LL – left lateral; LM – left medial; PiB-ve – PiB negative; PiB+ve – PiB positive; RL – right lateral; RM – right medial; ROI – region of interest.
