# Supplementary figures and images for: Differential effects of Down's syndrome and Alzheimer's neuropathology on default mode connectivity
Source: Hum Brain Mapp. 2019 Jul 26;40(15):4551–63. doi: 10.1002/hbm.24720 (PMC6865660; doi:10.1002/hbm.24720)

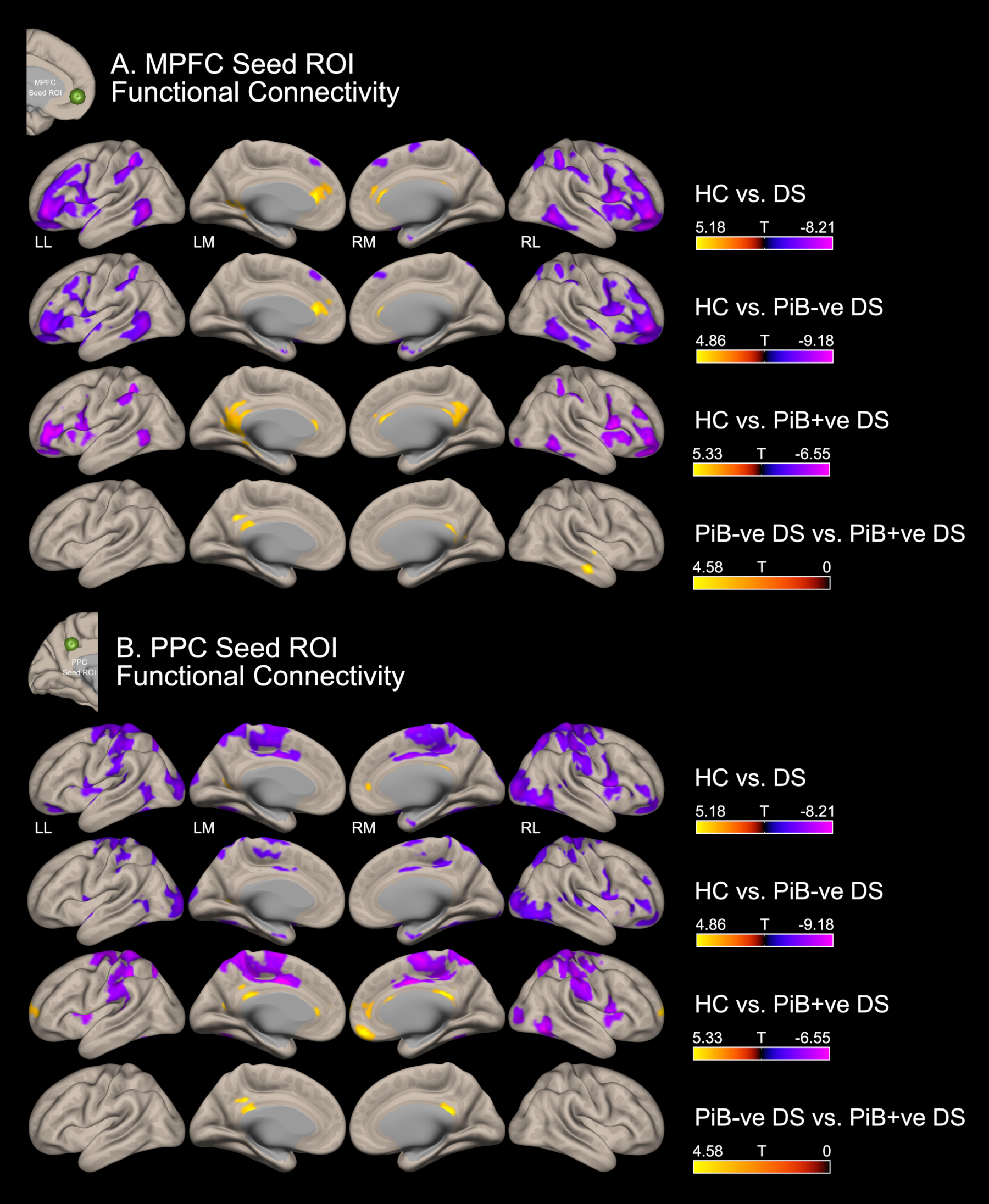

Supplement: Supplementary file 2 — Figure S1 Between groups differences in default mode network connectivity based on a medial prefrontal cortex (mPFC) seed is compared to group differences in default mode connectivity based on a posterior parietal cortex (PPC) seed. The location of the seed region in each analysis is shown in green. HC, healthy control; DS, Down's syndrome; LL, left lateral; LM, left medial; PiB −ve, PiB‐negative; PiB +ve, PiB‐positive; RL, right lateral; RM, right medial; ROI, region of interest. [file HBM-40-4551-s001.tif]
